# Cocaine

**Published:** 1886-01

**Authors:** M. C. Armstrong

**Affiliations:** Brookville, Indiana


### CORRESPONDENCE.

### Cocaine.

*Editor Dental Register:*

DEAR SIR:—With your permission and the privilege of a little space in your columns, I wish to reply to the article of Dr. C. C. Burns, of Greensburg, Indiana, published in the November number of the DENTAL REGISTER, in which he covertly, but none the less intentionally attacks me, on the uses of cocaine for dental purposes.

Dr. Burns says, "We want facts, not fancies." I gave *only facts*, in the article which he attempts to ridicule and make light of, by using my expression of "Eureka," and having the same italicised; and the fact, that these facts have been continually growing, and that my practice, with the use of this very beneficient anæsthetic, is constantly growing and expanding, until it has reached even into the Doctor's own city, is probably the chief cause of his somewhat bitter attack on the use of cocaine, "and more especially on the operator who cries eureka."

The Dr. says: "Had they given us the number of their failures, their little success would be left in the shade." I have now used cocaine in my practice continuously since last December, and in this period of more than eleven months I have not had a *single* failure.

I was called into the country a few weeks since, and at one farm house I used cocaine upon seven different persons, and extracted fifty-three teeth; and on a recent Saturday, in my office,

I extracted seventy-three teeth, administering cocaine to twelve different persons, neither of these patients suffering the slightest pain. And the affidavits of these, and many other citizens of this community, who have tested the truly wonderful merits of cocaine, can be furnished in substantiation of these "*facts*, not *fancies*," and among this number will be found leading members of the legal and medical professions, county officials, merchants, and farmers.

Neither do I believe, "That imagination goes a great ways, nor that locality has anything to do with the soothing effects which cocaine produces," nor do I think that Dr. Burns believed this when he wrote it, but that he inserted it merely for *buncombe.*

The Dr. refers, no doubt with some pride, to his "twenty-nine years of successful practice in dental surgery," and adds, "can it be *possible* that I know so little; are my perceptive faculties so deficient that I am unable to perceive the *eureka* that is contained in cocaine?"

History informs us that Sir William Pitt became a member of the English Parliament at the age of *twenty-one* years, also that he became Prime Minister and one of the brightest men in England at the age of *thirty*; hence there may be room for some doubt as to the value of these *long years of experience.*

Dr. C. C. Burns was my preceptor in the study of dentistry, and as I have always had a kindly feeling toward him, I was somewhat astonished upon reading his article. However, I am perfectly satisfied with my success in the practice, and in the use of cocaine, I have stated nothing but facts, and I have neither the time nor the inclination to carry on a journalistic controversy with my friend Dr. Burns; I shall continue the use of cocaine for dental purposes in my practice so long as it produces the good results that it has for me during the past eleven months, this attack of of my preceptor, to the contrary, notwithstanding, and with good feeling toward all members of the profession, this controversy ends here, so far as I am concerned. Respectfully,

M. C. Armstrong.

Brookville, Indiana.

